# Kawasaki Disease and COVID-19

**DOI:** 10.31138/mjr.31.3.268

**Published:** 2020-09-21

**Authors:** Athanasios Gkoutzourelas, Dimitrios P. Bogdanos, Lazaros I. Sakkas

**Affiliations:** 1 Department of Rheumatology and Clinical Immunology, Faculty of medicine, School of Health Sciences, University of Thessaly, Larissa; 2 Faculty of Medicine, School of Health Sciences, University of Thessaly, Larissa, Greece

**Keywords:** Atypical Kawasaki disease, Kawasaki-like disease, Kawasaki-COVID-19, paediatric inflammatory multisystem syndrome temporally associated with SARS-CoV-2, macrophage activation syndrome, myocarditis, toxic shock syndrome

## Abstract

The recent passing away of Dr. Tomisaku Kawasaki, who first described what is now known as Kawasaki Disease (KD), and recent reports of a multisystem inflammatory disease in children associated with the severe acute respiratory syndrome coronavirus 2 (SARS-CoV-2) (MIS-C), makes a review on KD and MIS-C timely. Kawasaki Disease is a systemic vasculitis with predilection for coronary arteries occurring mostly in early childhood. The main features are high fever, extensive skin rash, cheilitis with red, cracking, bleeding lips and strawberry tongue, conjunctivitis, erythema and induration of hands and feet, subsiding with periungual peeling, cervical lymphadenopathy, and coronary artery dilation/aneurysms. Treatment consists of intravenous (IV) immunoglobulin (Ig) plus acetylsalicylic acid. MIS-C is considered a cytokine storm with high fever, inflammation, multi-organ dysfunction, that shares features with KD, toxic shock, and macrophage activation syndrome. Many children require admission to paediatric intensive care units for circulatory support. Bacterial sepsis, staphylococcal toxic shock syndrome, and enterovirus-causing myocarditis should be excluded. Treatment is not standardized and includes IVIg, IV methylprednisolone and IL-6 and IL-1 inhibitors.

## INTRODUCTION

The recent passing away of Dr. Tomisaku Kawasaki who first identified a distinct disease entity now known as Kawasaki disease (KD), coincided with the COVID-19 pandemic and the reports of COVID-19-associated KD.

Dr. Tomisaku Kawasaki was a Japanese paediatrician in a Tokyo hospital when he saw a child with fever, red eyes, red rash, and desquamation of fingers and toes in 1961. The next year, he saw six more children with the same constellation of symptoms and signs. Being an astute physician, and recognising it as a distinct entity, he reported his findings in Japanese meetings, but was met with scepticism: he finally published a case series of 50 patients with acute febrile muco-cutaneous lymph node syndrome in 1967 in the Japanese Journal of Allergy.^1^ In the 1970s, KD was shown to cause coronary artery aneurysms.^1^

## KAWASAKI DISEASE

Kawasaki disease (KD) is an acute inflammatory disease characterised by medium-sized vasculitis with predilection for coronary arteries, predominantly affecting children <5 years of age. Although it may be a self-limited febrile illness, it is the most common cause of acquired heart disease in childhood in Japan, North America, and Europe.^2,3^ Furthermore, coronary artery aneurysms (CAAs) from KD affect adult life, accounting for 5% of acute coronary syndromes in individuals <40 years of age.^4^

The disease is more prevalent in Japan, with the highest recorded incidence rate of 264.8 per 100,000 children aged <5 years old in 2012. In the United States, children of Asian and Pacific Islander ancestry had the highest incidence rates among all racial groups, whereas white children had the lowest incidence (13.7/100000 children <5 years of age per year).

### Pathogenesis

The cause of KD is unknown, but it is generally accepted that viral agents can trigger the disease, as seasonal peaks of the disease coincide with seasonality of common respiratory infections. In countries in the Northern Hemisphere, the disease peak occurs in winter.^5,6^

Genetic factors appear to be involved in KD pathogenesis, as suggested by the increased susceptibility of KD in Asian children, as well as in children with Asian ancestry living in North America. In Japan, siblings of children with KD are at increased risk of developing the disease.^7^ Single nucleotide polymorphisms (SNPs) in multiple genes have been associated with increased susceptibility to KD including inositol 1,4,5 triphosphate kinase-C (ITPKC), caspase-3 (CASP3), FCGR2A, B cell lymphoid kinase (BLK), CD40, ORAI1, MBL, HLA class II.^5,8^ A twin study reported a concordance rate in monozygotic (MZ) 14% with no difference from dizygotic (DZ) twins, a finding which suggests that genetic factors probably do not contribute.^9^ However, it should be noted that twin studies are fraudulent with difficulties because subclinical coronary artery vasculitis may be missed in MZ twins.^10^ Elevated levels of interleukin (IL)-1, IL-6, and tumour necrosis factor (TNF)α as well as high levels of circulating neutrophils indicate involvement of innate immunity.^11^ Adaptive immunity is also involved, as both proinflammatory Th17 and regulatory T cells are found elevated in the circulation of patients with KD.^12^

Kawasaki disease vasculitis begins with neutrophil infiltration of the arterial wall that occurs during the first 2 weeks of the disease, and can destroy the vascular wall and cause aneurysms. Thereafter, there is lymphocytic infiltration with CD8+T cells, plasma cells, and monocytes, releasing pro-inflammatory cytokines IL-1β and TNFα, that may continue for months to years in a few patients (chronic arteritis).^8,13^ A proliferation of myofibroblasts in the arterial wall during the chronic phase leads to vascular lumen stenosis. Large saccular aneurysms with a thin wall consisted only of adventitia can rupture during the first 2–3 weeks from disease onset. Large aneurysms undergo sequential thrombosis, giving a false appearance of resolving.^13^ Reduced platelet microRNA (miR)-223 induction in KD may cause coronary pathology via miR-223/platelet growth factor receptor (PDGFR) β pathway.^14^

### Manifestations

The disease begins abruptly with high fever, and the first day of fever is considered the first day of the disease. The fever is remittent and lasts 1–3 weeks. Certain manifestations of KD are considered principal clinical manifestations and are included in the diagnostic criteria for the disease.

Diffuse and extensive maculopapular erythematous rash, extensive erythroderma, or erythema multiforme-like, appears in the first few days of the disease and is often transient. Severe psoriasis-like lesions with plaques and pustules may develop.^15^ Beau lines (transverse grooves in nails) may develop during the follow up period 1–2 months after the onset of the disease.

Painful swelling of hands and feet is a characteristic sign, with erythema of palms and soles that subsides with periungual peeling (desquamation). The latter occurs after day 10 and is considered a pathognomonic sign.^16^ Oral changes include cheilitis with red, cracking, bleeding lips, strawberry tongue, and oropharyngeal erythema. Retropharyngeal phlegmon may occur.

Bilateral conjunctival injection sparing the limbus without exudate is also a characteristic feature.

Cervical lymphadenopathy, usually unilateral, is seen in 60% of patients and is usually associated with retropharyngeal erythema.^17,18^

Coronary dilatations become evident after the first week of the disease. Pericarditis occurs in 18%, whereas myocarditis in much less frequent (3%), occasionally leading to cardiovascular shock.^19,20^

Other manifestations may originate from multiple organs/systems including gastrointestinal system (abdominal pain, diarrhoea, vomiting, hepatitis, gallbladder hydrops), genitourinary track (pyuria, phimosis), eyes (uveitis, retinal vasculitis), respiratory track (lung infiltrates). Arthritis develops in the first week of the disease. Neurological involvement is reported in 5% of patients and is manifested with headache, convulsions, irritability, aseptic meningitis, facial nerve palsy, and sensorineural hearing loss.^21^ Mediastinal lymphadenopathy is rarely reported.^22^ Some of these manifestations, such as pneumonia, nephritis, arthritis, myositis, uveitis and retinal vasculitis are considered atypical of KD.^16^

Infants and children >10 years old impose a difficulty in diagnosis of KD, and are at increased risk of CAAs.^16,23,24^ Infants may not manifest the full spectrum of disease manifestations and may have pyuria or irritability, whereas older children may have delayed manifestations.

### Laboratory Findings

Laboratory findings of KD are non-specific but may assist clinical evaluation of the patients. Typically, KD patients have increased levels of C-reactive protein (CRP), erythrocyte sedimentation rate (ESR), as well as white blood cell count with granulocyte predominance during the acute phase. Thrombocytosis usually is found after the first week of the disease. Less frequently, elevated levels of serum transaminases or gamma-glutamyl transpeptidase are found. Hypoalbuminemia is also a common finding, while mild hyperbilirubinemia is less frequent. Pyuria is common, and cerebrospinal fluid may show high cell count. In case of arthritis, synovial fluid shows an extremely high white cell count, with negative culture. Electrocardiogram abnormalities may be seen during the acute phase of the disease, while echocardiography is necessary to detect heart and coronary artery abnormalities. However, while echocardiography is sensitive in evaluating coronary artery abnormalities in small children, computed tomographic angiography and cardiac MRI are much more sensitive tools in older children.^5,25^

### Diagnosis

There is no diagnostic test for KD, and diagnosis depends on clinical criteria. According to the 2017 American Heart Association, criteria diagnosis requires fever for at least 5 days plus the presence of at least 4 of the 5 principal clinical features (**Table 1**).^5,25^

**Table 1.** The 2017 American Heart association criteria for Kawasaki disease.

Fever for at least 5 days plus ≥4 of 5 principal clinical features
1. Erythema and cracks on lips, strawberry tongue, and/or erythema of oral and pharyngeal mucosa
2. Bilateral bulbar conjunctival injection without exudate
3. Maculopapular rash, or diffuse erythroderma or multiforme-like erythema
4. Erythema and oedema of hands and feet in the acute phase and/or periungual desquamation in the subacute phase
5. Cervical lymphadenopathy, usually unilateral.
The presence of coronary artery aneurysms in patients with principal clinical features of KD confirms the diagnosis of KD.

Kawasaki disease is characterized as classic KD (also known as complete or typical KD) when the ≥4 principal clinical criteria are met. Such a diagnosis of classic KD can be made with 3 or 4 days with fever. Patients who have fewer principal clinical features may be considered for incomplete KD. The presence of coronary artery aneurysms confirms the diagnosis of KD.

The diagnosis of incomplete (or atypical) KD occurs mostly in infants and should be considered in any infant or child with prolonged unexplained fever, fewer than 4 of the principal clinical findings, and compatible laboratory or echocardiographic findings.^5^ The presence of coronary artery dilatations with Z-scores >2.5 for the left coronary artery of the right coronary artery is highly specific (specificity 98%) for KD.^26^

Differential diagnosis of KD includes viral infections, such as adenovirus, enterovirus, respiratory syncytial virus, metapneumovirus, coronaviruses, parainfluenza or influenza viruses, staphylococcal and streptococcal toxin-mediated diseases, drug hypersensitivity reactions, systemic onset juvenile idiopathic arthritis, Leptospirosis, and rickettsial infections.^5^ However, the diagnosis of KD should be considered when there are typical clinical features of KD, even in the presence of a documented infection. Wu et al. using serum levels of IL-17 (cut off value, 11.55 pg/mL) and NT-proBNP (cut off value, 255.5 pg/dL) reported a sensitivity of 86% and a specificity of 95% for differentiating incomplete KD from infectious diseases.^27^

Kawasaki disease lymphadenopathy should be differentiated from bacterial lymphadenitis. KD lymphadenopathy is usually multiple and associated with retropharyngeal oedema whereas bacterial lymphadenitis is single. Ultrasound (U/S) and CT scan can differentiate KD lymphadenopathy from bacterial lymphadenitis.^28^

Exudative pharyngitis, exudative conjunctivitis, oral ulcers, bullous or petechial skin lesions are not manifestations of KD, and their presence suggest alternative diagnoses

### Management

Intravenous immunoglobulin (IVIg) at a dose of 2g/Kg as a single infusion over 10–12 hours plus acetylsalicylic acid (ASA) are the standard treatment in KD used to reduce inflammation and prevent arterial damage.

The timely IVIg infusion in the acute phase of the disease reduces the risk for development of coronary artery abnormalities from 25% to ∼4%.^5,29–32^ Disease with fever persisted >36 hours after the IVIg infusion is considered resistant to IVIg. It should be noted that ESR may be increased by IVIg.

High dose of ASA (30–50 mg/Kg/day divided into 4 doses) is used during the acute phase until 48–72 hours after cessation of fever. Then, low dose of ASA (3–5 mg/Kg/day) is used for its antiplatelet effects until week 8 of the disease but indefinitely in patients with coronary artery abnormalities.^5,33^ ASA should be substituted by another antiplatelet agent for 6 weeks after varicella vaccination to prevent Reye syndrome.

About 10–15% of KD patients will be resistant to IVIg. These patients are also at increased risk of developing coronary artery abnormalities. In such cases, a second IVIg infusion (2g/kg) is usually given together with a tapering dose of corticosteroids and/or etanercept. Corticosteroids (IV pulse 2 mg/Kg/day for 5 days, followed by oral tapering over 2–3 weeks) are added to standard treatment in selected cases, and may decrease long-term coronary artery abnormalities.^5^

In patients refractory to the initial IVIg infusion, a systematic review showed that infliximab was more effective than a second IVIg and IV methylprednisolone in fever resolution.^34^ In a double-blind multicentre trial, etanercept used as adjunctive treatment after IVIg infusion reduced IVIg resistance in patients >1year of age and decreased coronary artery dilatation in patients with baseline coronary artery abnormalities.^35–39^

In severe cases of KD resistant to IVIg, additional treatments may include cyclosporine, anakinra, cyclophosphamide or even plasma exchange or combination of anakinra with etanercept.^5,40^ In cardiovascular shock, diuretics and vasopressors are utilized.

A particular complication relating to coronary artery aneurysms is thrombosis. Low-dose ASA is regularly used in all patients until week 6–8. In patients with rapidly expanding coronary artery aneurysms, low-molecular weight heparin (LMWH) or warfarin plus low-dose ASA is suggested, or triple treatment with LMWH, low-dose ASA, and a second antiplatelet agent (clopidogrel) may be considered.^5^ However, a systematic review showed insufficient evidence for the effectiveness of antiplatelet treatment in KD due to insufficient data and heterogeneity of studies.^41^

For occlusive or near-occlusive coronary artery thrombosis, thrombolytic treatment with tissue plasminogen activator (tPA, Alteplace 0.5 mg/Kg/hour over 6 hours) is used.

Long-term management of patients with KD after the end of the acute phase at week 4 to 6 involves thrombosis prophylaxis in patients with coronary artery abnormalities and regular evaluation of coronary arteries. Risk stratification for coronary artery abnormalities has been included in the 2017 AHA guidelines.^5^

## KAWASAKI DISEASE AND COVID-19: A COVID-19-INDUCED CYTOKINE STORM

The recent viral pandemics led support to the viral aetiology of KD. During the influenza H1N1 pandemic in December 2009, there was a sharp increase in KD cases.^42^ Similarly, the severe acute respiratory syndrome coronavirus-2 (SARS-CoV-2), a cause of the coronavirus pandemic in 2019–2020 (COVID-19), was associated with sharp increase in the incidence of KD.^42–44^ It should be noted that SARS-CoV-2 can infect endothelial cells and cause endothelial cell damage and thrombosis. In children presenting with chilblains, skin biopsy showed endothelial cell damage, thrombosis and SARS-CoV-2 in endothelial cells.^45^ SARS-CoV-2 binds to angiotensin converting enzyme 2 and excessively activates the angiotensin II pathway and then STING, a cytosolic DNA sensor and adaptor protein in type I IFN and nuclear factor(NF)-κB pathway, leading to hyper-coagulopathy via macrophage production of tissue factor.^46^

SARS-CoV-2 was initially reported as affecting children only mildly according to the Chinese Centre for Disease Control and Prevention, in an epidemiologic survey including 2135 SARS-CoV-2-exposed children and confirmed in other studies.^47–50^ Later, during the progression of the SARS-CoV-2 pandemic in Europe and USA, more and more case series were published reporting outbreaks of a severe multisystemic inflammatory syndrome in children exposed to SARS-CoV-2. This syndrome with multi-organ dysfunction, including high frequency of myocarditis, has been called multisystem inflammatory syndrome in children (MIS-C) by the World Health Organization (WHO) and the Centre for Disease Control (CDC),^51^ and shared features with staphylococcus aureus toxin-mediated toxic shock syndrome^52^ and atypical KD.^53,54^ It has been called paediatric multisystem inflammatory syndrome-temporally associated with SARS-CoV-2 (PIMS-TS) by the Royal College of Paediatrics and Child Health in UK.^55–57^

MIS-C is a cytokine storm induced by COVID-19 virus with very high inflammation markers (CRP, procalcitonin, neutrophilia, lymphopenia), pro-inflammatory cytokine levels, mainly IL-6, and high IL-10, soluble IL-2 receptor, ferritin levels, and D-dimers^58,59^ and frequently fulfils criteria for macrophage activation syndrome (MAS) of children with juvenile idiopathic arthritis.^60^

Studies of this syndrome in different parts of the world during the COVID-19 pandemic revealed similar findings and a frequent requirement for admission to paediatric intensive care unit (PICU). Riphagen et al reported 8 children presented in a south London UK hospital during a period of just 10 days with hyperimmune syndrome and shock, exhibiting features of atypical KD, KD shock syndrome. Five patients were SARS-CoV-2-positive/exposed. Clinical features were high fever (38–40°C), rash, conjunctivitis, peripheral oedema, abdominal pain, diarrhoea, vomiting, and cardiovascular shock requiring haemodynamic support. No significant respiratory involvement was noted. Laboratory findings included high CRP, procalcitonin, ferritin, Troponin, NT-proBNP, and D-dimers, and low platelet count.^43^ A similar report from Verdoni et al. in the province of Bergamo, Italy, during the SARS-COV-2 epidemic compared 10 patients (aged 7.5 years [SD 3.5]) diagnosed with KD between Feb 18 and April 20, 2020 (group 1), with 19 KD patients (aged 3.0 years [SD 2.5]), diagnosed between Jan 1, 2015 and Feb 17, 2020 (group 2).^44^ In group 1, eight of 10 patients were SARS-CoV-2 positive, six of 10 had cardiac involvement, five of 10 had KD-shock syndrome, and five of 10 patients had MAS. These patients had a more severe disease course with resistance to IVIg and need of adjunctive steroids.^44^

A multisystem inflammatory syndrome in children (MIS-C) associated with infection with SARS-CoV-2 was reported in children from paediatric health centres across the United States and the New York area at the time of SARS-CoV-2 pandemic.^53,54,61^ This hyperimmune serious and life-threatening syndrome in children and adolescents affected many organs/systems including gastrointestinal, cardiovascular, haematological, mucocutaneous, and respiratory, with features of both KD and toxic shock syndrome. Many patients were admitted to the intensive care unit.^53,54^ Prominent gastrointestinal symptoms, left ventricular systolic dysfunction with elevation of IL-6, and NT pro-BNP and macrophage activation, as well as other similar findings were also reported in children from France and Switzerland.^62,63^

Paediatric inflammatory multisystem syndrome-temporally associated with SARS-CoV-2, a hyperinflammatory syndrome with features of KD and toxic shock syndrome, has been described in children from France and the UK. The syndrome differed from KD by the high frequency of myocarditis (44%), low platelet count, and high frequency of IVIg resistance. Cardiac involvement in PIMS-TS was very frequent and included elevated Troponin I, CK, pro-BNP, coronary abnormalities, reduced ejection fraction, transient valve regurgitation and ECG abnormalities. Patients usually required inotropes and/or vasopressors. Inflammatory markers were highly elevated, including CRP, ferritin, and procalcitonin.^55,56^

The clinical spectrum of MIS-C overlaps with KD, but there are notable differences between the two. Patients with MIS-C have a broad age range from early childhood to late adolescence, whereas KD predominantly occurs in early childhood, and lymphopenia, cardiac ventricular stress including myocarditis, lymphopenia and thrombocytopenia are common in MIS-C. Coagulopathy is also an important feature in MIS-C. Patients with MIS-C may have 1–4 features of KD, whereas coronary artery aneurysms can develop in MIS-C patients without KD features. The immunologic features of MIS-C resemble MAS, but not very closely. IL-6, IL-10, sIL-2R are elevated in MIS-C but IL-18 and IFNγ are mildly elevated, whereas ferritin, although elevated, is lower than in MAS.^59^ IL-1 was not elevated in a case series. It should be noted that IL-1 and Il-18 are markedly elevated in MAS.^61^

Bacterial sepsis, staphylococcal and streptococcal toxic shock syndrome, as well as infections causing myocarditis, such as enterovirus, should be excluded.^57^

There is no standard treatment for MIS-C. Many patients received IVIg usually with methylprednisolone 1–4 mg/Kg/day, while IL-6 or IL-1 inhibitors may be used (20%).^53–55,59,61,63^ Few patients received Remdesevir.^59^ Anti-coagulation with low-dose ASA plus enoxaparin is also used.^59^

In conclusion, the multisystemic inflammatory syndrome in children (MIS-C) related to the SARS-CoV-2 pandemic (also termed Kawasaki-like disease, or Kawa-COVID-19) appears to share clinical, pathogenetic and laboratory features with KD, toxic shock syndrome, and MAS. The most important differences between the MIS-C and KD are the greater age of disease onset, more frequently gastrointestinal involvement, myocarditis and/or cardiogenic shock and heart failure requiring inotropic support, circulatory assistance and PICU admission. Also, MIS-C may have resistance to IVIG infusion treatment. MIS-C is a cytokine storm driven predominantly by IL-6 and IL-8 while in patients with KD, IL-1 appears to be the main mediator of coronary artery inflammation.
